# Recombinant Human Endostatin Normalizes Tumor Vasculature and Enhances Radiation Response in Xenografted Human Nasopharyngeal Carcinoma Models

**DOI:** 10.1371/journal.pone.0034646

**Published:** 2012-04-09

**Authors:** Fang Peng, Zumin Xu, Jin Wang, Yuanyuan Chen, Qiang Li, Yufang Zuo, Jing Chen, Xiao Hu, Qichao Zhou, Yan Wang, Honglian Ma, Yong Bao, Ming Chen

**Affiliations:** 1 Department of Radiation Oncology, Sun Yat-Sen University Cancer Center, Guangzhou, Guangdong Province, China; 2 State Key Laboratory of Oncology in South China, Guangzhou, Guangdong Province, China; 3 Cancer Center, Affiliated Hospital of Guangdong Medical College, Zhanjiang, China; 4 Organ Transplantation Center, The First Affiliated Hospital, Sun Yat-Sen University, Guangzhou, Guangdong Province, China; 5 Guangzhou General Hospital of Guangzhou Military Command, Guangzhou, Guangdong Province, China; Medical College of Wisconsin, United States of America

## Abstract

**Background:**

Hypoxic tumor cells can reduce the efficacy of radiation. Antiangiogenic therapy may transiently “normalize” the tumor vasculature to make it more efficient for oxygen delivery. The aim of this study is to investigate whether the recombinant human endostatin (endostar) can create a “vascular normalization window” to alleviate hypoxia and enhance the inhibitory effects of radiation therapy in human nasopharyngeal carcinoma (NPC) in mice.

**Methodology/Principal Findings:**

Transient changes in morphology of tumor vasculature and hypoxic tumor cell fraction in response to endostar were detected in mice bearing CNE-2 and 5–8F human NPC xenografts. Various treatment schedules were tested to assess the influence of endostar on the effect of radiation therapy. Several important factors relevant to the angiogenesis were identified through immunohistochemical staining. During endostar treatment, tumor vascularity decreased, while the basement membrane and pericyte coverage associated with endothelial cells increased, which supported the idea of vessel normalization. Hypoxic tumor cell fraction also decreased after the treatment. The transient modulation of tumor physiology caused by endostar improved the effect of radiation treatment compared with other treatment schedules. The expressions of vascular endothelial growth factor (VEGF), matrix metalloproteinase-2 (MMP-2), MMP-9, and MMP-14 decreased, while the level of pigment epithelium-derived factor (PEDF) increased.

**Conclusions:**

Endostar normalized tumor vasculature, which alleviated hypoxia and significantly sensitized the function of radiation in anti-tumor in human NPC. The results provide an important experimental basis for combining endostar with radiation therapy in human NPC.

## Introduction

The survival, growth, and metastasis of solid tumors are dependent on tumor blood vessels [1], [2]. Tumor vessels are both structurally and functionally abnormal compared with normal vessels, bearing defective endothelium, basement membrane and pericyte coverage. Blood vessels are immature, tortuous, dilated, and saccular, and have a haphazard pattern of interconnection. These structural abnormalities contribute to spatial and temporal heterogeneity in tumor blood flow, thus impairing the delivery of oxygen [2], [3], [4].

Hypoxia is a common phenomenon in solid tumors and is associated with aggressive clinical phenotype and poor prognosis [5], [6]. Resistance of hypoxic tumor cells to radiation is a significant reason of failure in the local control of tumors, especially the squamous cell carcinomas. Over 50 years ago Thomlinson and Gray discovered the resistance of hypoxic cells to radiation therapy, since then researchers have been attempting to eliminate tumor hypoxia, but there is no effective approach [7].

Since one of the major factors causing hypoxia is the inadequate vascular supply of tumor, more recent attempts to improve tumor radiation response have involved specifically targeting the tumor blood supply. However, this strategy also seems counterintuitive: it is a widely held belief that antiangiogenic therapy eradicates tumor vasculature, thus depriving the tumor of oxygen and nutrients. However, preclinical models have shown that antiangiogenic therapy may transiently “normalize” the tumor vasculature to make it more efficient for oxygen delivery, thereby providing a window of opportunity for enhanced sensitivity to radiation treatment [3], [8], [9], [10], [11], [12], [13], [14], [15], [16]. Since there are no guidelines for optimal scheduling of antiangiogenic therapy and radiation therapy, the timing of the administration of antiangiogenic agents, relative to the delivery of ionizing radiation, would be critical for optimizing the antitumor effect of these treatments.

Nasopharyngeal carcinoma (NPC) is fairly rare among Caucasians in Western Europe and North America, but there is a higher incidence in Southeast Asia. NPC is sensitive to radiotherapy. Radiotherapy or combined chemoradiation therapy is the mainstay treatment modality. Now distant metastasis remains a major cause of death [17]. Advances in radiation and chemotherapy have also improved the prognosis of individuals with NPC. However, patients still may suffer from the disease as a result of local-regional therapeutic resistance, which results from tumor hypoxia in NPC. It therefore makes sense to improve treatment efficacy for patients with NPC [18]. Recently, tumor vascular normalization was found in several solid tumors, but such role of antiangiogenic agents in NPC remains unknown.

Endostatin, a 20 kD C-terminal fragment of collagen XVIII, is an endogenous inhibitor of angiogenesis [19]. Endostar, traditional endostatin with an additional nine-amino acid sequence at the N-terminal of the protein and a six-histidine tag, were reported to be more efficient in blocking angiogenesis and suppressing primary tumor and metastasis growth [20]. Endostar was approved by the State Food and Drug Administration (SFDA) for the treatment of non-small cell lung cancer (NSCLC) in 2005. Phase III and IV clinical studies have verified that combining endostar with standard chemotherapy regimens could successfully improve the median survival time and overall survival rate of patients with advanced NSCLC [21]. However, the effects of endostar combined with radiotherapy have been unclear.

We hypothesized that endostar could normalize the morphology and function of NPC vasculature for a period of time, leading to transient improvement in tumor oxygenation and response to radiation therapy. Here, we investigated transient changes in morphology of tumor vasculature and hypoxic tumor fraction in response to endostar in human NPC in mice. We evaluated the effects of endostar combined with radiation therapy in human NPC and also explored potential mechanisms.

## Materials and Methods

### Ethics statement

All animal work was conducted according to relevant national and international guidelines. The animal use protocol has been reviewed and approved by the institutional animal care and use committee of Sun Yat-Sen University (IACUC SYSU, NO.20100703004). For details please refer to subsection entitled Cell culture and animal model.

### Cell culture and animal model

Male BALB/c nude mice, aged 5–6 weeks, were purchased from the Shanghai Laboratory Animal Center of Chinese Academy of Sciences (Shanghai, China). Mice were allowed to acclimate to local conditions for at least 1 week and maintained under a 12-hours dark, 12-hours light cycle with food and water *ad libitum*. All of the experiments were carried out in accordance with guidelines approved by the Laboratory Animal Center of Sun Yat-sen University. Two poorly differentiated nasopharyngeal carcinoma cell lines (CNE-2 and 5–8F) [22], [23] (conserved in our laboratory) were maintained in RPMI 1640 medium supplemented with 10% fetal bovine serum, 100 units/ml penicillin G, and 100 ng/ml streptomycin at 37°C in 5% CO_2_ [24], [25]. Cells were diluted to a concentration of 10^7^/mL in phosphate buffer solution (PBS); mice were injected subcutaneously with 0.1 ml of the suspension into the right back flank.

### Radiation delivery

Local irradiation of the implanted tumor was administered using a customized mouse jig with other parts of the body shielded with lead. Each mouse was confined to a customized mouse jig with a circular window, through which the tumor bed was exposed to the radiation and irradiated locally. Mice were exposed to X-rays with 5-mm thick lead shields when the tumor bed was gently extended into the radiation field. Tumors were locally irradiated with 6 Gy of 160 kV X-rays using RS 2000 X-ray Biological Irradiator (Rad Source Technologies, USA) at a dose rate of 1Gy/min through a 0.2-mm copper filter.

### Treatments

The treatments were initiated when tumors reached a size of about 100 mm^3^. Control animals were injected with 0.9% saline only. For the treated groups, endostar (20 mg/kg/d) diluted in 0.9% saline was administered subcutaneously. Endostar was provided by Simcere Pharmaceutical Research Co., Ltd. When the treatment modalities were combined, mice bearing CNE-2 tumor were randomly assigned to six treatment groups (n = 6–8 per group): untreated control, endostar alone (20 mg/kg/d for 10 days from the first day of treatment), radiation alone (6 Gy), and radiation in combination with endostar (20 mg/kg/d for 10 days from the first day of treatment), which radiotherapy was delivered at day 3, day 5, or day 9 after the first endostar injection. Again, mice bearing 5–8 tumors were randomly assigned to five treatment groups as we combined the treatment modalities (n = 6–8 per group): untreated control, endostar alone (20 mg/kg/d for 10 days from the first day of treatment), radiation alone (6 Gy), and radiation in combination with endostar (20 mg/kg/d for 10 days from the first day of treatment), which radiotherapy was delivered at 3 or 7 days after the first endostar injection. Tumor volume was measured by using a caliper every other day and calculated according to the following formula: V = L×W^2^/2 (L, length; W, width). Body weights of mice were monitored as an indirect measurement of general toxicity.

### Whole-mount staining for tumor vascularity

Primary tumors were double stained using a whole-mount staining protocol for CD31 and type IV collagen/NG2 [26], [27] (n = 4–6 animals per group). Sections of tissues were fixed in 4% formalin overnight, cut at a thickness of 60 µm. Sections dried on superfrost plus slides were permeabilized with PBS containing 0.3% triton X-100, and treated with proteinase K (20 µg/ml). Specimens were incubated for 1 hour in a blocking solution containing 4% goat serum, and then incubated with primary antibodies at 4°C over night. Endothelial cells, vascular basement membrane, and pericytes of tumor vessels were identified by staining with combinations of two antibodies. Endothelial cells were labeled with rat monoclonal anti-CD31 (PECAM-1, clone MEC 13.3; 1∶500; Biosciences-Pharmingen). Vascular basement membrane was examined with rabbit polyclonal anti-type IV collagen antibody (1∶2,000; Chemicon). Pericytes were labeled with rabbit anti-mouse NG2 antibody (1∶400; Chemicon). Tumor tissues were double-stained with an anti-CD31 antibody and an anti-type IV collagen or anti-NG2 antibody. Tissues were rinsed in PBS containing 0.3% triton X-100, and then incubated for 2 hour at room temperature with secondary antibodies. Secondary antibodies were goat anti-rat Alexa Fluor 555–conjugated antibodies (1∶500; Invitrogen) or goat anti-rabbit dylight 488–conjugated antibodies (1∶500; Jackson ImmunoResearch). Sections were rinsed in PBS containing 0.3% Triton X-100, and mounted in anti-fade reagent.

### Confocal scanning microscopy quantifications

Tissue sections were examined under a confocal microscope (Olympus Confocal FV1000 Microscope). 2D images of each tissue sample were assembled. Image acquisition was performed maintaining the same laser power, gain, and offset settings. Positive signals were photographed under a fluorescent microscope (20× objective magnification). In each mouse, 9–12 digital fluorescence microscopic fields of view were analyzed randomly and captured in a region measuring 512×512 µm. Images were analyzed using ImageJ software. Endothelial cells (red channel: CD31), basement membrane (green channel: type IV collagen), and pericytes (green channel: NG2) of blood vessels in tumors were quantified by measuring the proportion of sectional area (area density). Based on an analysis of pixel fluorescence intensities, which ranged from 0 to 255, specific staining was distinguished from background by empirically using a threshold value of 40 or 50. Area densities of structures stained with CD31, type IV collagen or NG2 were calculated as the proportion of pixels having a fluorescence intensity value equal to or greater than the corresponding threshold. We calculated the ratio of mean fluorescence intensity of green to red channel. Values are expressed as percentage of green to red co-staining.

### Immunofluorescent visualization of tumor hypoxia

Hypoxia in tumors was detected by the formation of pimonidazole adducts [9], [11]. Pimonidazole hydrochloride compound was injected intraperitoneally into each animal at a dose of 60 mg/kg. One hour after injection, mice were sacrificed, and tumors were dissected and immediately fixed in either 4% formalin or frozen. Frozen tumors were cut into 20 µm sections which were then immunostained and used to detect pimonidazole adducts by using Hypoxyprobe-1 Mab1 FITC Ab (Hypoxyprobe-1 Plus kit; Chemicon) in the whole tumor regions (n = 4–6 animals per group) following the manufacturer's instructions. The sample counterstained with DAPI at 1 µg/mL. Images of the sections were captured by using Olympus confocal FV1000 microscope at 200× magnification. The slides were imaged using a computer controlled scanning stage and later “stitched” together to gain one image of the entire tumor. We calculated the ratio of green (Pimonidazole) to blue (DAPI) channel mean fluorescence intensity.

### Immunohistochemistry for angiogenic factors

CNE-2 tumor-bearing mice were sacrificed 5 days after endostar treatment. Tumor tissues from each group were formalin-fixed and embedded in paraffin, then sectioned at 5 µm thickness and stained with H&E according to standard immunohistochemical procedures. The sections were used to detect the expression of vascular endothelial growth factor (VEGF), pigment epithelium-derived factor (PEDF), matrix metalloproteinase-2 (MMP-2), MMP-9, and MMP-14. The samples were incubated with rabbit polyclonal VEGF (1∶200; Abcam), rabbit polyclonal PEDF (1∶200; Chemicon), rabbit anti-mouse polyclonal MMP-2 (1∶50; Santa Cruz), rabbit polyclonal MMP-9 (1∶50; Labvision), rabbit polyclonal MMP-14 (1∶50; Thermo). Primary antibody diluent was used as a negative control in all IHC studies. The immunoreactivity positive cells from each of the differently treated tumor tissue sections were measured at 200× magnification using a light microscope. The amount of proteins was analyzed by integral optical density (IOD) using IPP (Image Plus Pro 6.0, Bethesda, MD, USA).

### Statistics

Means and SDs were calculated for continuous variables. For two-group comparison, the t-test method was used. For more than two groups' comparison, one-way ANOVA was used firstly to detect the differences amongst these groups. If the P-value was less than 0.05, multiple comparison was performed using LSD-t test. Statistical analyses of results were performed using the standard 2-tailed Students t test by SPSS software version 13.0 (SPSS Inc.). *p*<0.05 was considered statistically significant.

## Results

### Tumor vasculature

**Figure 1:**
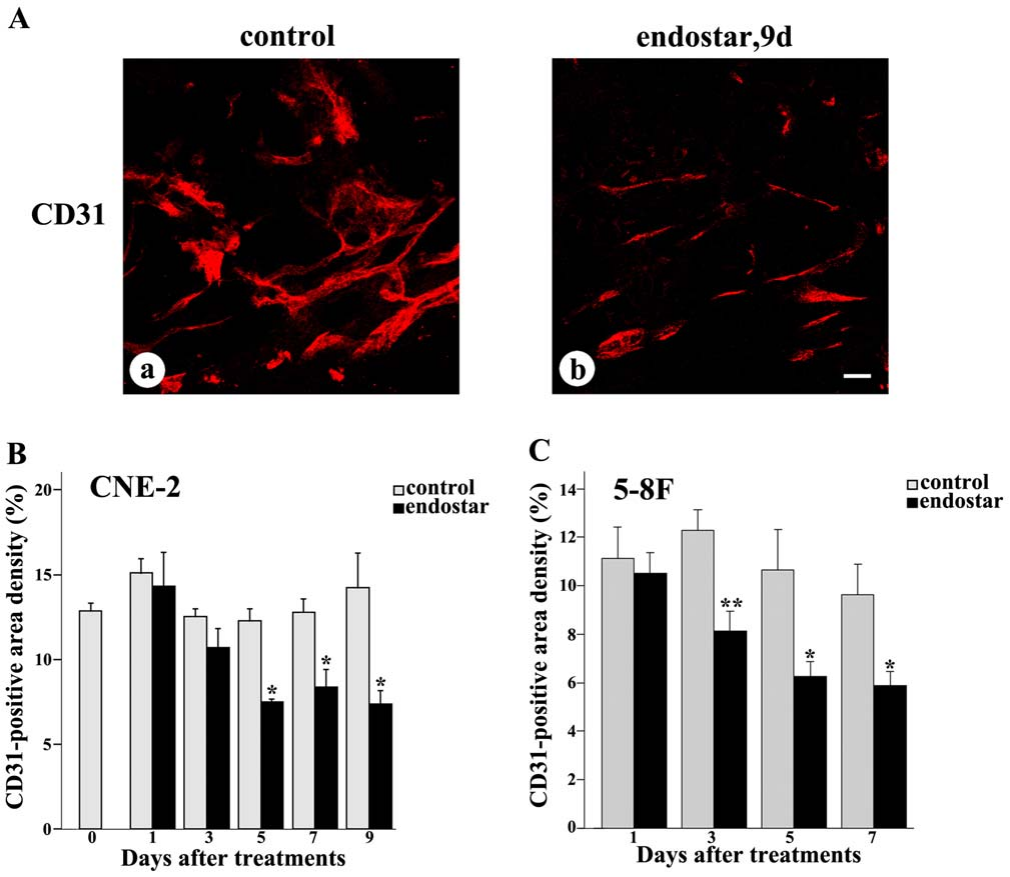
Regression of tumor vasculature after endostar treatment. CNE-2 and 5–8F tumors were removed and stained with anti-CD31 antibody. Tumor blood vessels were analyzed by confocal microscopy at different days after treatment as indicated. Tumor blood vessels are presented in red. A-a: CD31 had an irregular distribution in control tumors after 9 days of treatment with vehicle in CNE-2 tumor. A-b: After the treatment with endostar for 9 days, reduction in CD31 in CNE-2 tumor was reflected by decreased immunofluorescence. B–C: Quantification of CD31-positive tumor vessels. Bar graphs illustrated changes in area density of CD31-positive vessels in CNE-2 and 5–8F tumors. Quantificatoin were determined from 9–12 randomized cryosectioned fields (n = 4–6 mice per group). Columns, means; bars, SEM. *p<0.05 compared with the control group, * *p<0.01 compared with control (Student's t-tests). Scale bar: 50 µm.

Immunofluorescent staining with antibody directed against CD31 was used to investigate whether the tumor vascularization and organization were modified after endostar treatment. Blood vessels in untreated CNE-2 tumors were abundant, tortuous, and variable in diameter (Fig. 1A-a), while tumor vessels that survived after 9 days of treatment with endostar were less irregular, less tortuous, more uniform in caliber, and had fewer branches and sprouts (Fig. 1 A-b). Measurements of the vascularity of the tumors showed a conspicuous reduction in CD31 immunoreactivity after 5, 7, or 9 days of treatment with endostar in CNE-2 xenografts (Fig. 1B) and after 3, 5, or 7 days in 5–8F xenografts (Fig. 1C). Overall vascularity decreased by 48% during 9 days of endostar treatment in CNE-2 xenografts and 39% during 7 days of endostar treatment in 5–8F xenografts.

### Vascular basement membrane

**Figure 2:**
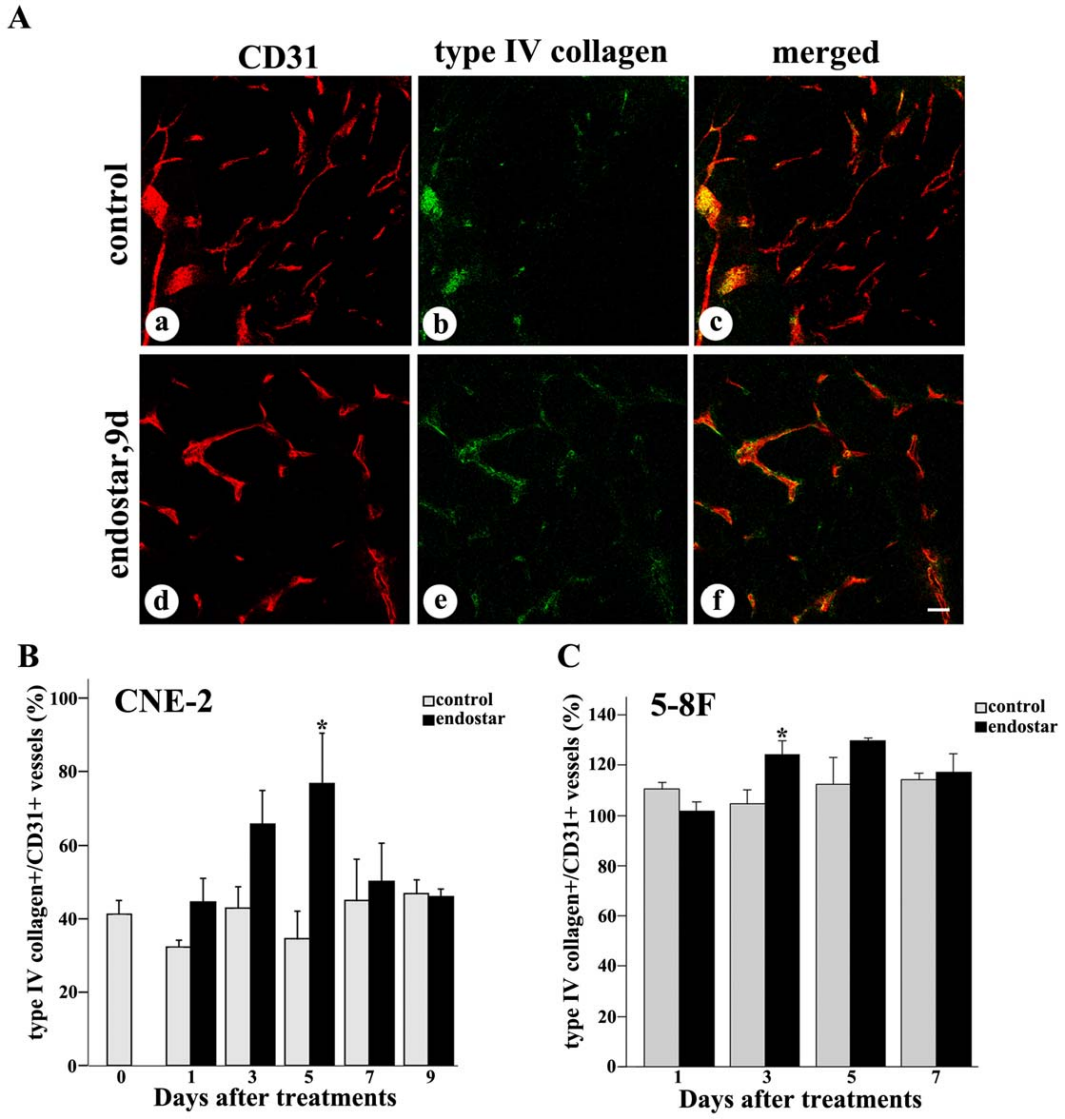
Confocal microscopic images showing comparison of vascular basement membrane in NPC tumors with or without endostar treatment. Fluorescence images of tumors showed CD31-positive endothelial cells (red), type IV collagen-positive basement membrane (green), and merged images (orange). A: Some segments of CD31 lacked type IV collagen immunoreactivity in untreated CNE-2 tumors (a–c). Most CD31 coincided with regions of type IV collagen-positive blood vessels 5 days after endostar treatment in CNE-2 tumors (d–f). B–C: Graphs of the percentage of type IV collagen showed that basement membrane was increased during regression of endothelial cells in both CNE-2 and 5–8F tumors. Quantification of type IV collagen-positive vessels versus total CD31-positive vessels were determined from 9–12 randomized cryosectioned fields (n = 4–6 mice per group). Columns, means; bars, SEM. *p<0.05 compared with the control group, * *p<0.01 compared with control (Student's t-tests). Scale bar: 50 µm.

Using CD31 and type IV collagen immunoreactivities as markers, we compared the fates of endothelial cells and vascular basement membrane of blood vessels during blood vessel regression. Under baseline conditions, distinct strands of CD31 unaccompanied by type IV collagen staining indicated the absence of basement membrane (Fig. 2A a–c). By comparison, the conspicuous loss of CD31-positive vessels was not accompanied by a corresponding reduction in type IV collagen after the treatment with endostar. Capillaries had a continuous basement membrane as reflected by type IV collagen and CD31 immunoreactivities (Fig. 2A d–f). The treatment with endostar did significantly increase the percentage of basement membrane sleeves in CNE-2 tumors (Fig. 2B), while a little increase was found in 5–8F tumors (Fig. 2C). The percentages of type IV collagen immunoreactivity in control and endostar-treated tumors after the 5 day treatment were 34.6% and 76.7% respectively.

### Pericyte coverage

**Figure 3:**
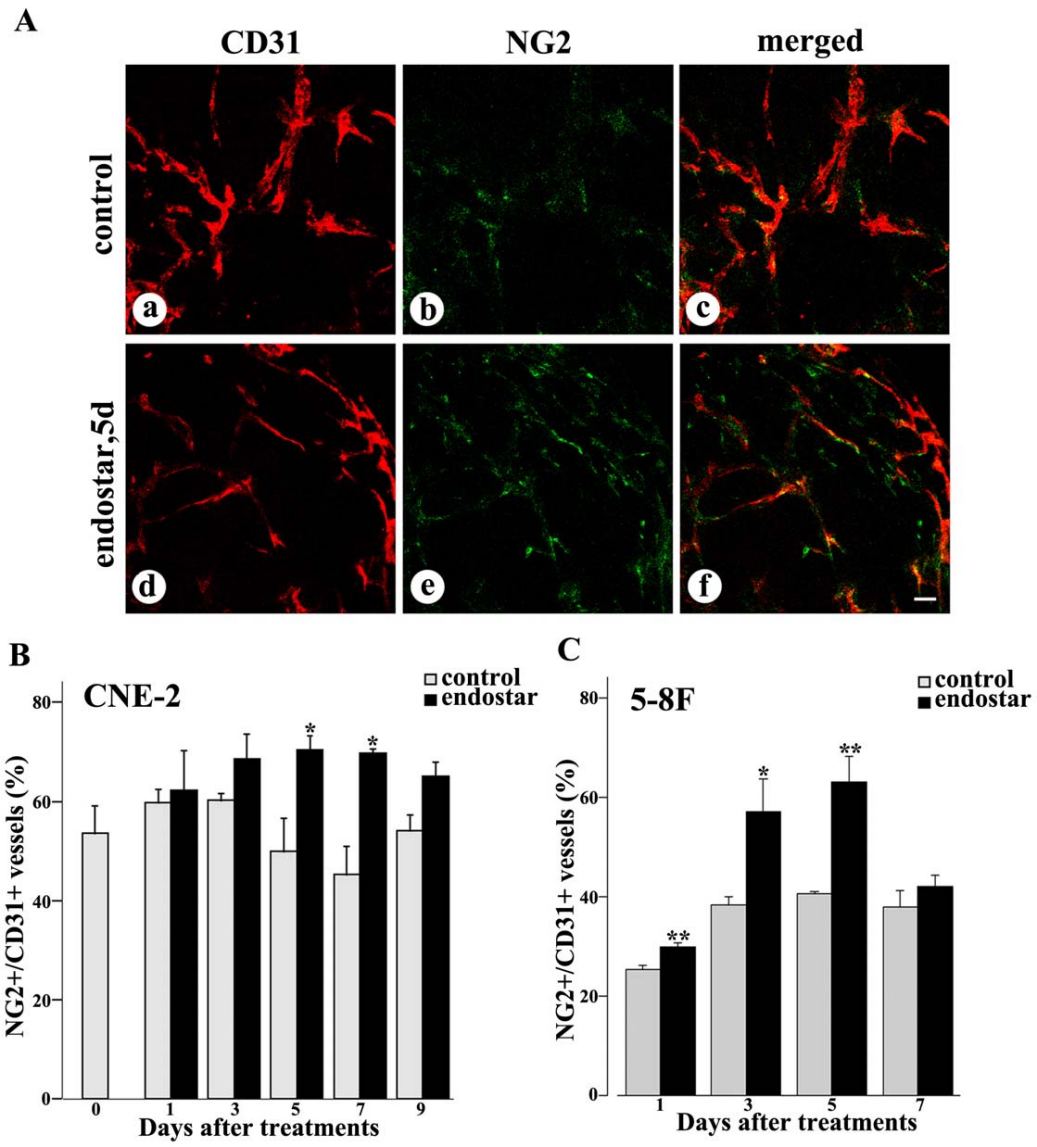
Confocal microscopic images showing comparison of pericyte coverage in NPC tumors with or without endostar treatment. Fluorescence images of tumors showed CD31-positive endothelial cells (red), NG2-positive pericytes (green), and merged images (orange). A: Some segments of CD31 lacked NG2 immunoreactivity in untreated CNE-2 tumors (a–c). The intensity of NG2 immunofluorescence colocalized with CD31 staining were increased 5 days after endostar treatment in CNE-2 tumors (d–f). B–C: Graphs of the percentage of NG2 showed that pericytes were increased during regression of endothelial cells in both CNE-2 and 5–8F tumors. Quantification of percentages of NG2-positive vessels versus total CD31-positive vessels was determined from 9–12 randomized cryosectioned fields (n = 4–6 mice per group). Columns, means; bars, SEM. *p<0.05 compared with the control group, * *p<0.01 compared with control (Student's t-tests). Scale bar: 50 µm.

Increased pericyte coverage was considered as a key characteristic of tumor vessel normalization. Accordingly, we analyzed the pericyte coverage of tumor blood vessels after the treatment with endostar. The expression of molecular markers that define specific subpopulations of pericytes (NG2) was studied. We observed that endostar substantially increased the pericyte coverage of tumor blood vessels in CNE-2 xenografts after the treatment with endostar for 5 days (Fig. 3A d–f), compared to the untreated tumors (Fig. 3A a–c). After 5 days treatment with endostar, ratios of NG2 immunoreactivity to CD31 immunoreactivity were increased by 42% in CNE-2 xenografts and 56% in 5–8F xenografts, respectively (Fig. 3B–C). Yet pericyte NG2 expression returned to baseline by day 9 in CNE-2 xenografts and day 7 in 5–8F xenografts after endostar treatments.

### Overal tumor oxygenation

**Figure 4:**
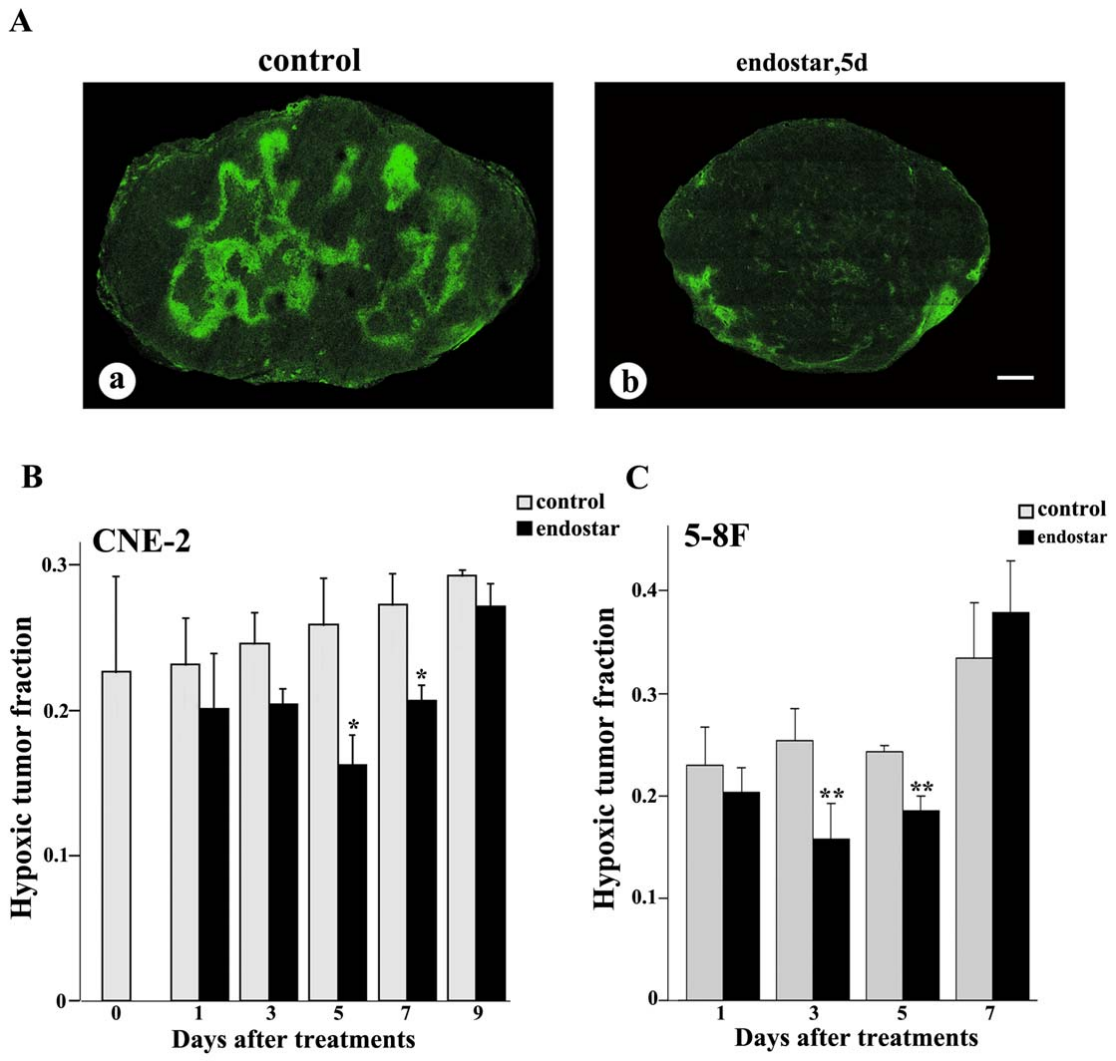
Confocal microscopic images showing comparison of tumor hypoxia in NPC tumors with or without endostar treatment. Tumor hypoxia (pimonidazole staining, green) was severe in control tumors (A-a), but decreased on day 5 during monotherapy with endostar (A-b) in CNE-2 tumor. Hypoxia reached a minimum at day 5, and a partial relapse occurred at day 9 in CNE-2 tumor (B), while hypoxia reached a minimum at day 3, and a relapse occurred at day 7 in 5–8F tumor (C). Quantification of hypoxic tumor fraction was determined from the whole tumor cryosectioned fields (n = 4–6 mice per group). Columns, means; bars, SEM. *p<0.05 compared with the control group, * *p<0.01 compared with control (Student's t-tests). Scale bar: 1 mm.

To explore whether treatment with endostar resulted in an increase in overall tumor oxygenation, we used pimonidazole staining to evaluate the hypoxic fraction of both CNE-2 and 5–8F xenografted tumors. Our findings demonstrated that the hypoxic tumor fraction was significantly decreased on day 5 after the treatment with endostar (Fig. 4A-b) compared with the saline-treated mice in CNE-2 xenografts (Fig. 4A-a). During the treatment of endostar, tumor hypoxia began to drop on day 1, decreased by 59% on day 5, and increased again by day 9 in CNE-2 xenografts (Fig. 4B). Similarly, it dropped on day 1, decreased by 38% on day 3, and increased again by day 9 in 5–8F xenografts (Fig. 4C).

### Tumor growth

**Figure 5:**
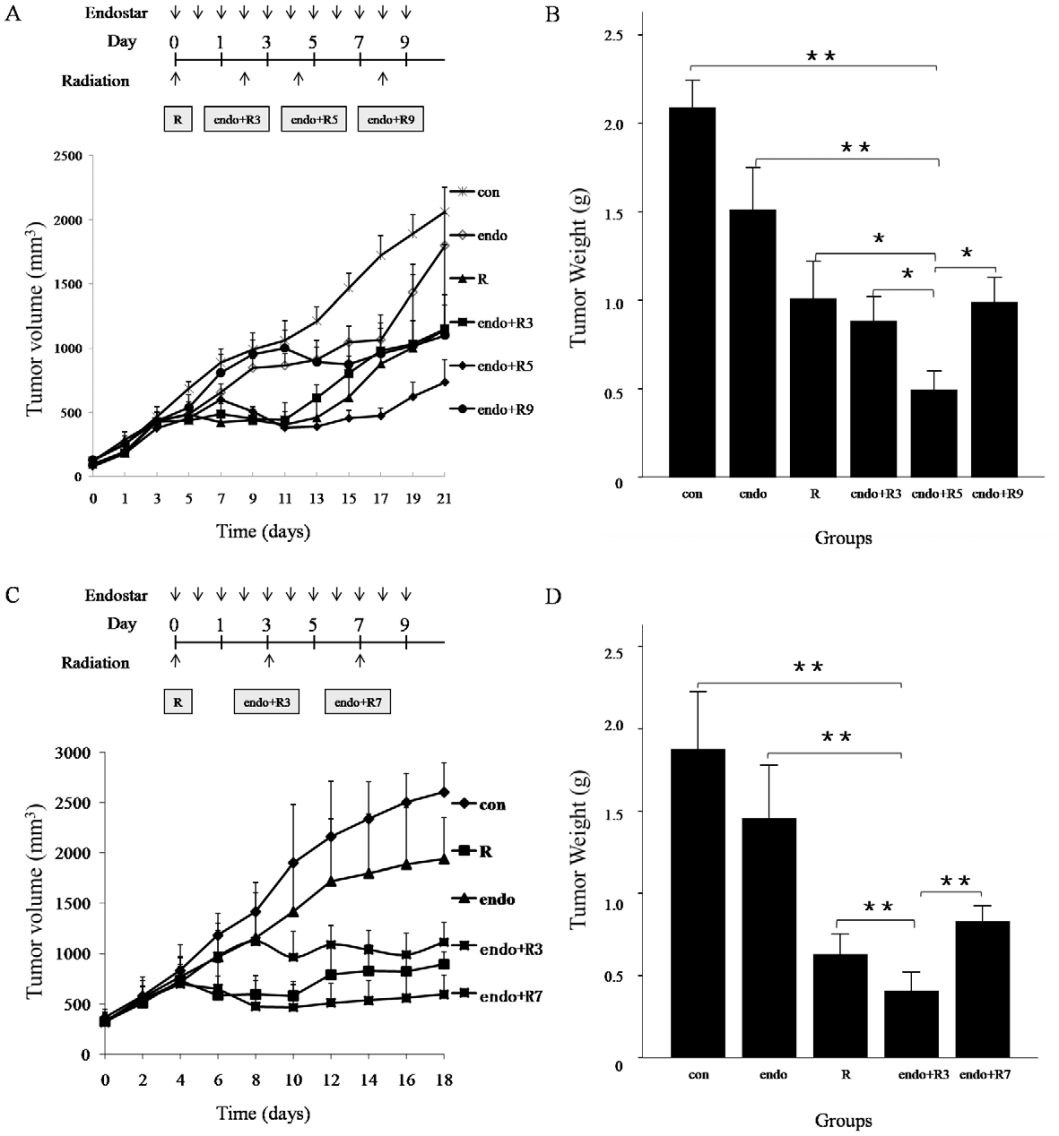
Effect of the combination of endostar and radiation on NPC tumor growth. Subcutaneously implanted CNE-2 and 5–8F xenografted tumors were established as described in Materials and Methods. When xenografted tumors reached a volume of 100 mm^3^, mice in CNE-2 xenografts were randomly divided into six groups (n = 6–8 per group): con, control (0.9% saline); endo, endostar only; R, radiation at a single dose of 6 Gy only; endo+R3, radiation at a single dose of 6 Gy on the 3^rd^ day after endostar treatment; endo+R5, radiation at a single dose of 6 Gy on the 5^th^ day after endostar treatment; endo+R9, radiation at a single dose of 6 Gy on the 9^th^ day after endostar treatment. Mice in 5–8F xenografts were randomly divided into five groups (n = 6–8 per group): con, control; endo, endostar only; R, radiation at a single dose of 6 Gy only; endo+R3, radiation at a single dose of 6 Gy on the 3^rd^ day after endostar treatment; endo+R7, radiation at a single dose of 6 Gy on the 7^th^ day after endostar treatment. Endostar was administered at a dose of 20 mg/kg/d for 10 days from the first day of treatment. A, C: The growth curve of CNE-2 and 5–8F tumor xenografts. Radiation induced a significantly tumor suppression when combining with endostar on day 5 and day 3. B, D: A combination of radiation and endostar is synergistic only during a “normalization window” when tumor hypoxia is greatly diminished. Each point represents the mean tumor size. Columns, means; bars, SEM. *p<0.05 compared with the control group, * *p<0.01 compared with control (ANOVA, LSD-t test).

We evaluated the effects of radiation and/or endostar on tumor growth in vivo. Mice were randomly assigned to receive saline treatment (control), radiation alone (6 Gy), endostar alone, or radiation administered after 3, 5, 9 days treatment with endostar in CNE-2 tumors and 3, 7 days treatment with endostar in 5–8F tumors. We found that either endostar or radiation could modestly inhibit tumor growth compared with untreated controls (Fig. 5, *p*<0.05). In contrast, 5 days or 3 days after the treatment with endostar administration of radiation significantly reduced the CNE-2 or 5–8F tumor volumes compared with untreated control mice or mice treated with radiation or endostar alone (Fig. 5, p<0.05). When it was the maximum difference for the control, endostar-treated, radiation-treated, and radiation after 3, 5, 9 days treatment with endostar treated mice, tumor volumes in CNE-2 xenografts measured on day 17 were 1727±151, 1067±197, 880±176, 982±214, 473±64, 957±197 mm^3^, respectively. Endostar significantly enhanced the antitumor effect of radiation on the day 5 in CNE-2 xenografts and the day 3 in 5–8F xenografts. Animals treated with endostar (alone or in combination with radiation) showed no signs of toxicity as assessed by unaltered behavior, weight gain during experiments both in CNE-2 and 5–8F xenografts.

### Angiogenic factors

**Figure 6:**
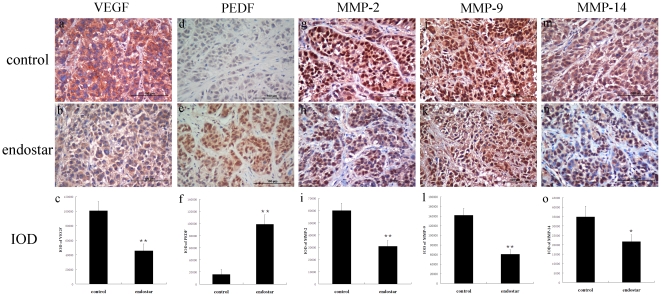
Immunohistochemical staining and quantitative analysis for VEGF, PEDF, MMP-2, MMP-9, and MMP-14 in human CNE-2 xenograft tissues. Xenografts were harvested at day 5 after treatment initiation. The levels of VEGF, MMP-2, MMP-9 and MMP-14 were decreased, while the level of PEDF was increased in CNE-2 xenografts treated with endostar for 5 days compared with the control group (Student's t-tests). a–c, VEGF staining and quantitative data; d–f, PEDF immunostaining and quantitative data; g–i, MMP-2 immunostaining and quantitative data; j–l, MMP-9 immunostaining and quantitative data; m–o, MMP-14 immunostaining and quantitative data. n = 6/group. *p<0.05 compared with control, * *p<0.01 compared with control (Student's t-tests). Scale bar: 100 µm.

To further investigate whether vascular normalization was associated with VEGF/PEDF balance and MMPs which were involved in remodeling of the extracellular matrix, we used immunostaining to assess the effects of endostar on the expression of VEGF, PEDF, MMP-2, MMP-9 and MMP-14 in CNE-2 xenografted tissues. Our study suggested that VEGF were decreased while PEDF were increased and concentrated in CNE-2 tumor cells 5 days after endostar treatment. Meanwhile, the expression levels of MMP-2, MMP-9 and MMP-14 were significantly decreased by endostar (Fig. 6).

## Discussion

Preclinical and clinical studies have showed that anti-angiogenic agents could normalize tumor vasculature. Winkler et al. demonstrated that VEGFR-2 (VEGF receptor-2) blockade DC101 reduced the mean basement membrane thickness while the subcutaneously implanted mammary carcinomas had incomplete vascular basement membrane coverage [9]. Furthermore, VEGFR-2 blockade DC101 has been reported to increase basement membrane coverage to near normal levels. In addition, many other anti-angiogenic agents, such as DC101, bevacizumab, trastuzumab, erlotinib, TNP-470, gleevec, erbitux, sunitinib, TSU68, and KRN951, can normalize the tumor vascular system in a variety of animal models [9], [11], [16], [28], [29], [30], [31].

To explore whether endostar normalized the tumor vasculature in NPC, we established the models of human CNE-2 and 5–8F nasopharyngeal carcinoma xenografts in nude mice and treated the mice with endostar as described in Materials and Methods. Our observation showed that blood vessels in the endostar-treated tumors were less irregular, less tortuous (Fig. 1). Furthermore, the results indicated that the percentages of basement membrane and pericyte coverage were significantly increased by endostar (Fig. 2–3), which was thought to be a key property of vessel normalization [32]. For all these reasons, our data demonstrated that endostar could create a vascular normalization time window from day 5 to day 7 after treatment in CNE-2 NPC xenografts and day 3 to day 5 after treatment in 5–8F NPC xenografts.

Vascular normalization alters intratumoral vascular physiology by lowering interstitial fluid pressure, reducing vessel permeability, and thus decreasing intratumoral hypoxia [13], [33]. Consequently, we further evaluated the effect of endostar on the hypoxic condition of human NPC xenografts. Our data showed, in agreement with the previous studies, that tumor hypoxic fraction decreased on day 5 to day 7 in CNE-2 xenografts and day 3 to day 5 in 5–8F xenografts after endosar treatment (Fig. 4), which suggested that intratumoral hypoxia was improved during the endostar treatment.

Mounting evidence supports the view that alleviation of tumor hypoxia and improved vascular delivery may enhance the cytotoxic effects of chemotherapeutics and ionizing radiation [8]. Our data showed that the radiation response was the greatest when radiation was administered on the day 5 in CNE-2 xenografts and day 3 in 5–8F xenografts after endostar treatment, whereas the other time windows showed no benefits (Fig. 5), which was consistent with the previous studies. Bevacizumab, an anti-VEGFR antibody, has been demonstrated to induce tumor vascular normalization and enhance the anti-tumor effects of ionizing radiation in heterotopic models. Dings et al. recently reported that radiation therapy delivery was most effective during a “tumor oxygenation window” after treatment with bevacizumab in heterotopic models of human ovarian carcinoma, murine melanoma, and breast carcinoma [11]. Meanwhile, bevacizumab has been demonstrated to improve intratumoral oxygenation and enhance the antitumor activity of ionizing radiation in glioma [13]. Preliminary results of a phase II clinical study of chemoradiation plus bevacizumab for locally/regionally advanced NPC (RTOG 0615) suggested that bevacizumab prolonged overall survival [34]. Therefore, the results of the present study are of translational importance for not only clarified that endostar could create a vascular normalization time window after treatment in NPC xenografts, but aslo showed that the anti-tumor effect of irradiation was enhanced during the normalization time induced by delivering endostar.

Interestingly, the combination of radiation with endostar showed enhanced anti-tumor effects, which can be attributed to the local decrease in tumor hypoxia and the increase in oxygenation. However, at day 3 and day 9 the addition of endostar lacks even an additive benefit to radiation therapy in CNE-2 xenografts. One possibility was the transient nature of vascular normalization after endostar treatment, which was likely due to expression of alternative pro-angiogenic factors by tumors [35]. However, the underlying mechanisms need to be further investigated.

Malignant tissues produce multiple angiogenic factors to induce neovascularization, which significantly contributes to tumor growth and metastasis. Of all the known angiogenic molecules, VEGF (vascular endothelium growth factor) appears to be the most critical. VEGF promotes the survival and proliferation of endothelial cells and increases vascular permeability [36]. VEGF is overexpressed in the majority of solid tumors and down-regulating VEGF signaling might normalize tumor vasculature [32]. Some studies provide evidence that severe abnormalities of the vascular basement membrane in tumor vessels are mediated by VEGF signaling, since VEGFR-2 blockade restores a thinner, more closely attached basement membrane monolayer [9]. Pigment epithelum-derived factor (PEDF), an endogenous anti-angiogenic and anti-tumor factor [37], is more potent than any other known endogenous inhibitors of angiogenesis, being more than twice as potent as angiostatin and seven times more potent than endostatin [38]. Furthermore, VEGF/PEDF ratio is a key molecular mechanism for antiangiogenic therapy against malignant diseases. Gao et al. reported that plasminogen kringle 5 could down-regulate VEGF and up-regulate PEDF thus to decrease the VEGF/PEDF ratio, and lead to the restoration of a normal balance between angiogenic stimulators and inhibitors, which might be the mechanism for the anti-angiogenic activity of plasminogen kringle 5 [39]. In the present study, we found that VEGF was down-regulated by endostar, while PEDF was up-regulated (Fig. 6). We speculated that during the normalization window, endostar increased pericyte coverage of NPC tumor vessels via upregulation of PEDF and downregulation of VEGF, thus inhibiting VEGF singaling.

MMPs, a family of extracellular and membrane-associated endopeptidases, collectively digest almost all extracellular matrix and basement membrane components. They play a crucial role in various physiological processes including tissue remodeling and organ development and thus in tumor progression [40]. Recent studies have identified MMPs as modulators of the tumor microenvironment [41]. MMP-2, MMP-9, and MMP-14 have been reported to be involved in tumor angiogenesis and extracellular matrix remodeling. Our study provided evidence that endostar decreased MMP-2, MMP-9, and MMP-14 expression, which could be responsible for reduced angiogenesis in treated CNE-2 NPC xenografts compared to controls (Fig. 6). This suggestion is supported by previous studies in which showed the involvement of these MMPs in tumor angiogenesis [42], [43]. However, the effects of MMPs on tumor vascular normalization remain unknown and further investigation is required. We presumed that the downregulation of MMP-2, MMP-9 and MMP-14 might alleviate the extracellular matrix (ECM) degradation, thus maintaining the vascular basement membrane and normalizing tumor vasculature.

In general, we concluded that endostar normalized tumor vasculature within a time window, alleviated hypoxia and significantly sensitized the antitumor effect of radiation in NPC xenograft models. We found that during the normalization window, endostar might increase pericyte coverage of NPC tumor vessels via upregulation of PEDF and downregulation of VEGF, thus inhibiting VEGF singaling. Our data implies that endostar might be a potent adjuvant therapeutic agent for NPC therapy in combination with radiotherapy. These results are of translational importance because the clinical benefits of endostar therapy might be increased by more precise treatment scheduling to ensure that radiation is given during periods of peak radiosensitivity.
